# Exploring the Associations between Functional Capacity, Cognitive Function and Well-Being in Older Adults

**DOI:** 10.3390/life12071042

**Published:** 2022-07-13

**Authors:** Pinelopi S. Stavrinou, George Aphamis, Marios Pantzaris, Giorgos K. Sakkas, Christoforos D. Giannaki

**Affiliations:** 1 Department of Life and Health Sciences, University of Nicosia, Nicosia 2417, Cyprus; aphamis.g@unic.ac.cy (G.A.); giannaki.c@unic.ac.cy (C.D.G.); 2 University of Nicosia Research Foundation, Nicosia 1700, Cyprus; 3 The Cyprus Institute of Neurology and Genetics, Nicosia 2371, Cyprus; pantzari@cing.ac.cy; 4 School of Physical Education and Sport Science, University of Thessaly, 42100 Trikala, Greece; gsakkas@med.uth.gr

**Keywords:** aging, cognition, executive function, quality of life, fatigue, sleep, strength, endurance, fitness

## Abstract

Background: The present study aimed to explore the associations between functional capacity and global cognition, executive function and well-being in older adults. Methods: Ninety-seven older adults (age 80.6 ± 8.2 years) were examined for global cognitive function (Mini-Mental State Examination), executive function (symbol cancellation test), functional capacity (sit-to-stand tests, 6 min walk test, timed up-and-go test and handgrip strength test) and well-being (quality of life, fatigue levels, sleep quality and daily sleepiness). Adjusted partial correlations were computed to examine the associations between variables. Mediation analyses were conducted to evaluate whether functional capacity would mediate the relationships between age and cognitive or executive function. Results: Greater levels of functional capacity were associated with better performance in cognitive and executive function tests (*p* < 0.05). Mediation analyses revealed that functional capacity partially mediated the effects of age on global cognition and executive function (indirect effect: β = −0.11, 95% CI = −0.20 to −0.03; β = 0.34, 95% CI = 0.13 to 0.57, respectively). Increased levels of functional capacity were also associated with higher quality of life (*p* < 0.05, r = 0.32 to 0.41), lower fatigue levels (*p* < 0.05, r = 0.23 to 0.37), and better sleep quality (*p* < 0.05, r = 0.23 to 0.24). Conclusions: Functional capacity can mediate the effects of age on global cognition and executive function in older adults and greater levels of functional capacity are associated with improved quality of life, better sleep quality, and lower fatigue levels.

## 1. Introduction

Population aging is a global phenomenon; in the European Union, the number of people aged 65 and over will become a much larger share, rising from 19.5% in 2016 to 29.2% of the population in 2070 [1]. For this reason, as this challenge emerges for health care systems, new directions of action must be generated in order to respond to the increasing aging-related health issues of the geriatric population.

Neurodegenerative diseases are emerging as a common medical condition in the geriatric population. Among them, dementia is the most prevalent disorder, which affects approximately 7 million people in the European Union and is estimated to double by 2050 [2]. The spectrum of cognitive decline in older adults ranges from what can be classified as normal cognitive decline with aging to subjective cognitive impairment (cognitive complaint with normal cognitive screening test) to mild cognitive impairment (MCI) to dementia [3]. Age-related cognitive decline is a robust predictor of mortality in older people, even when it does not progress to dementia [4]. Cognitive impairment has also been associated with an increased likelihood of adverse outcomes, including frailty [5], serious fall-related injury [6] and disability [7].

Besides cognitive impairment, another major cause of disability and dependency among older people is the deterioration of functional capacity that occurs with aging [8,9]. Importantly, this decline often constitutes the early stages of a continuous process, leading to the inability to effectively carry out activities of daily living [10]. Thus, maintenance of cognitive and functional independence is essential in older adults, as cognitive impairment and activities of daily living dependencies emerged as the strongest predictors of nursing home admission [11]. Although it is becoming increasingly apparent that functional capacity and cognitive function are interrelated [12,13], it is important to more fully understand the relationship between these two parameters in older adults and also how their impairment can affect well-being. Quality of life is a multidimensional construct that includes physical, mental, emotional, and social functioning domains. According to the Centers for Disease Control and Prevention (CDC) of the United States, assessing quality of life can help determine the burden of preventable disease, injuries, and disabilities, and can provide valuable new insights into the relationships between quality of life and risk factors [14]. Cognitive function and functional capacity are key metrics in older adults, both from the perspective they provide on aggregate health and as vital contributing factors to the ability of older adults to adequately perform activities of daily living and prevent a further decline in their quality of life. Notable reductions in quality of life, sleep disturbances and fatigue commonly occur with advancing age, with larger changes arising in the oldest old [15,16,17].

Although there are a growing number of studies examining the association between cognitive and physical function, many of them evaluate global cognition. Executive function is a cognitive domain that allows people to engage in independent and purposive behavior [18]. Executive abilities are described as high-level cognitive functions that include goal formation, planning, carrying out goal-directed plans, and effective performance [18] and are important to just about every aspect of life [19]. It is known that executive function is age-related and vulnerable to decline [20]. Evaluation of executive deficiencies has important clinical implications, since it has been suggested that executive dysfunction might be a predictor of subsequent development of Alzheimer disease in old age [21] and is a key contributor to impairment in activities of daily living [22,23]. In this regard, studies have presented associations between executive function and specific aspects of functional capacity, such as gait speed and balance [24,25]. However, associations between executive function and other parameters of functional capacity, such as aerobic fitness and upper and lower extremity strength, are less studied.

Therefore, as both functional capacity and cognitive function are important determinants of independent living, it is highly relevant from a clinical perspective to identify the relationship between these parameters and their impact on well-being. A better understanding of these associations could provide insight for developing interventions to support healthy aging. Engaging in physical activity and exercise has been shown to provide protection against age-related cognitive function decline through various mechanisms such as increased neurotrophins and cerebrovascular reserve and reduced inflammation [13,26]. Accordingly, it is interesting to evaluate whether an increased functional capacity could mediate the negative effect of aging on cognition. Furthermore, since it is also important to identify which functional capacity components are more relevant for older adults, we aimed to look at a broad range of functional capacity parameters, across the spectrum of normal cognition and cognitive impairment. Thus, the main aim of the present study was to examine whether functional capacity would mediate the relationship between age and cognitive function (global and executive). A secondary aim was to examine the associations between cognitive function, different functional capacity parameters, sleep quality, fatigue level and health-related quality of life among older adults.

## 2. Materials and Methods

### 2.1. Participants

This was a cross-sectional study designed to assess various aspects of functional capacity, cognitive function and well-being among older adults. Power analysis for partial correlation was conducted in G*POWER software to determine a sufficient sample size using an alpha of 0.05, a power of 0.80, a medium effect size of 0.3 and two tails. Based on the above assumptions, the desired sample size was 82. Participants were recruited from long-term care facilities, and community and medical centers from the two largest cities of the country (distance between the cities: 85 km). The exclusion criteria of the population were: (a) age < 65 years, (b) inability to walk and stand alone or with technical assistance, (c) inability to understand and answer the questionnaires, (d) presence of dementia (MMSE < 10) and (e) having severe visual or hearing impairment. The descriptive characteristics of the participants are presented in Table 1.

The participants’ demographic information and medical history were retrieved from medical records. The Charlson Comorbidity Index was used to evaluate medical comorbidity [27], with higher scores indicating greater comorbidity. All participants provided their informed consent to the study procedures. The study was conducted in accordance with the declaration of Helsinki and approved by the National Bioethics Committee (CNBC/RP/2017/24).

### 2.2. Study Overview

The participants were evaluated in two testing days by the same examiners to assess body composition, cognitive function, functional capacity, and well-being following the same testing order. During their first day of tests, the participants underwent a body composition evaluation. Afterwards, a cognitive function assessment was executed consisting of two tests: the Mini-Mental State Examination (MMSE) and the symbol cancellation test. During the second test day, well-being was assessed using questionnaires evaluating quality of life, fatigue, sleep quality and sleepiness. The interview method was used for the completion of all questionnaires. Then, the functional capacity measures were performed, including the timed up-and-go test (TUG), the handgrip strength test (HGS), the three sit-to-stand tests (STS-5, STS-30 and STS-60) and the 6 min walk test (6MWT). The tests were performed in the same order for all participants, allowing adequate time for recovery between each test. A lower number of participants was included in the analysis of some tests due to various reasons (e.g., inability to complete the test, health reasons).

### 2.3. Cognitive Function Assessments

#### 2.3.1. Mini-Mental State Examination (MMSE)

MMSE is the most widely used measure of global cognition [28]. It contains questions that have been grouped into categories representing a different domain or function: orientation to time (5 points); orientation to place (5 points); registration (3 points); attention and calculation (5 points); recall (3 points); language (8 points) and visual construction (1 point) [29]. The MMSE score has a range of 0–30 points, where a higher score indicates a better cognition level. Participants with an MMSE score lower of 10 were excluded from the study due to the inability to understand the procedures.

#### 2.3.2. Symbol Cancellation Test

Cancellation tests have been considered as a potential measure of executive function [30,31]. The symbol cancellation test provides a measure of neglect, visuospatial function, organizational process, and selective attention [30,32,33]. The participants were asked, in 45 s, to cross out 60 target symbols against a background of over 300 distractor symbols arranged randomly. Each target omission or incorrectly identified symbol was marked as 1 error [32]. The test was scored on the number of errors.

### 2.4. Functional Capacity Tests

#### 2.4.1. Timed Up-and-Go Test (TUG)

The TUG test examines balance, gait speed, and functional ability of an individual and it measures the time taken by the participant to stand up from a chair, walk a distance of 3 m, turn, walk back to the chair, and sit down again [34].

#### 2.4.2. Handgrip Strength Test (HGS)

HGS is a widely used indicator of overall muscle strength for aging adults [9]. Handgrip strength of the participants was measured using a digital dynamometer (T.K.K. 5401 Grip-D; Takey Scientific Instrument Co., Ltd., Tokyo, Japan). In a seated position, each individual was asked to squeeze the dynamometer as hard as possible for 3–5 s. Three measurements for each hand, alternating sides, were executed and the best of the grip strength measurements was reported as the final result.

#### 2.4.3. Sit-to-Stand Tests (STS-5, STS-30 and STS-60)

The sit-to-stand tests evaluate lower body power, balance and muscle endurance and require participants to rise from a chair to a full stand with their arms across their chest and then return back to the initial seated position [10,35]. The STS-5 test measures the time to perform five sit-to-stand cycles, while the STS-30 and STS-60 score is the total number of cycles completed in 30 and 60 s, respectively.

#### 2.4.4. 6 min Walk Test (6MWT)

The 6MWT evaluates the exercise capacity and endurance of the participants. The test requires the participant to cover as much ground as possible in six minutes and the total distance walked is measured [36].

### 2.5. Body Composition Assessments

Body mass and height were measured to the nearest 0.05 kg (Seca model 755, Hamburg, Germany) and 0.1 cm (Seca model 720, Hamburg, Germany), respectively. Body mass index (BMI) was calculated as body mass (kg) divided by height (m^2^). Total body fat percentage and trunk fat percentage were evaluated using bioelectrical impedance analysis (Bodystat 1500 and Viscan Tanita abdominal fat analyzer AB140, Tokyo, Japan).

### 2.6. Well-Being Questionnaires

#### 2.6.1. Quality of Life

The Short-Form 36 Health survey (SF-36) was used to evaluate quality of life [37]. The SF-36 is a 36-item questionnaire that produces scores on eight domains (physical functioning, role physical, bodily pain, general health, vitality, social functioning, role emotional and mental health), which are summarized in two components: the physical health and the mental health component. The total score ranges from 0% to 100% with higher scores indicating better functioning.

#### 2.6.2. Sleep Quality

Subjective sleep quality was evaluated with the Pittsburg Sleep Quality Index [38]. This questionnaire includes subscales to estimate subjective sleep quality, sleep latency, sleep duration, habitual sleep efficiency, sleep disturbances, use of sleeping medication, and daytime dysfunction over the past month (total score range 0–21; scores > 5 indicate poor sleep quality).

#### 2.6.3. Daily Sleepiness

The Epworth Sleepiness Scale was used for assessing the participants’ general level of daytime sleepiness [39]. This questionnaire consists of 8 self-rated items, each scored from 0 to 3, that measure a subject’s habitual likelihood of dozing or falling asleep in common situations of daily living. The score of this questionnaire is the sum of the eight item-scores and ranges from 0 to 24. Values > 10 indicate excessive daytime sleepiness.

#### 2.6.4. Fatigue

Fatigue levels were evaluated by using the fatigue severity scale (FSS) [40]. This 9-item scale assesses the effect of fatigue on daily living. The score has a range of 1–7, with lower scores indicating less fatigue. The final score represents the mean value of the 9 items.

Cronbach’s alpha coefficients were computed to estimate the internal consistency reliability for each quality of life scale and for the sleep quality, daytime sleepiness and fatigue questionnaires. Internal consistency for the quality of life questionnaire exceeded the 0.70 standard in all scales (ranging from 0.809 (mental health scale) to 0.940 (physical function scale)), except the social functioning and general health scales, which scored 0.686 and 0.684, respectively. Reliability scores for sleep quality, daytime sleepiness and fatigue questionnaires were 0.766, 0.809 and 0.933, respectively. The validity of the Greek versions of these questionnaires has been previously demonstrated [41,42,43,44].

### 2.7. Statistical Analysis

Statistical analysis was performed using SPSS version 22. Welch’s *t*-test was used for comparisons between sexes. Adjusted partial correlations for age, sex and education were also computed to examine the relationships between functional capacity, cognitive function and well-being. The magnitude of the correlations for r = 0.12, 0.20, and 0.32 was interpreted as small, medium, and large effects according to the guidelines for effect size in gerontology [45]. A composite functional capacity score was derived through the use of factor analysis by combining the six raw scores (STS-5, STS-30, STS-60, TUG, HGS and 6MWT). The raw scores loaded into a single factor explained 73.3% of total variation. The extraction method used was Maximum Likelihood, and the resulting functional capacity score is a standardized index where positive (negative) values are associated with higher (lower) functional capacity than the average functional capacity. In order to evaluate whether functional capacity would mediate the relationship between age and cognitive function, mediation analyses were conducted for both global cognition and executive function. For these mediation analyses, cognitive function was specified as the dependent variable with age specified as the independent variable and functional capacity as the mediator. In these analyses, mediation is significant if the 95% bias-corrected and accelerated confidence intervals (lower limit, upper limit) for the indirect effect do not include zero [46]. The analysis was performed using the PROCESS macro v.3.5 in SPSS v.22 using the model 4 Bootstrapping, with 5000 resampling iterations. Models were adjusted for sex and education. The Bonferroni correction method was used to adjust for multiple comparisons. *p* < 0.05 was considered statistically significant.

## 3. Results

Table 1 presents the sociodemographic characteristics, body composition evaluation, and performance on cognitive function and functional capacity tests of the participants. Ninety-seven participants were enrolled in the study, of whom 66% were female. Seventy-five percent (75%, *n* = 73) of the participants were residents of long-term care facilities (24 male and 49 female). According to their MMSE score, 41% (*n* = 40) of the participants were non-cognitively impaired (MMSE ≥ 24), while no significant difference was observed between the sexes in any of the cognitive function tests. Overall, 71% (*n* = 63/89) were obese (BMI ≥ 30 kg/m^2^), with females presenting higher values of total and trunk fat than males (*p* < 0.001). In functional capacity tests, the females had significantly lower performance than the males in all parameters (*p* < 0.05), except the 6MWT (*p* = 0.162).

The results of the well-being questionnaires’ data for all participants are presented in Table 2. Low scores of quality of life were observed in both sexes, with females presenting significantly lower values in physical and mental health components as well as in total score than males (*p* < 0.05). Sixty-eight percent of the participants (*n* = 66) showed low sleep quality and twenty-three percent (*n* = 22) presented high daily sleepiness. No significant differences were found in sleep quality, sleepiness and fatigue between the sexes (*p* > 0.05).

Table 3 reports the partial correlations between the measures of functional capacity, cognitive function parameters and well-being questionnaires after controlling for age, sex and education. All functional capacity parameters were significantly associated with both cognitive function measures (all *p* < 0.05, r from 0.23 to 0.49), indicating that better functional capacity results were reflected by a better cognitive performance. An exception was the association between TUG and symbol cancellation performance (r = 0.19, *p* = 0.087). Almost all functional capacity parameters were significantly associated with higher quality of life and lower fatigue levels (*p* < 0.05, r from 0.23 to 0.41). Sleep quality was also significantly correlated with some functional capacity parameters (6MWT, STS-30 and STS-60; all *p* < 0.05, r from 0.23 to 0.24).

**Figure 1:**
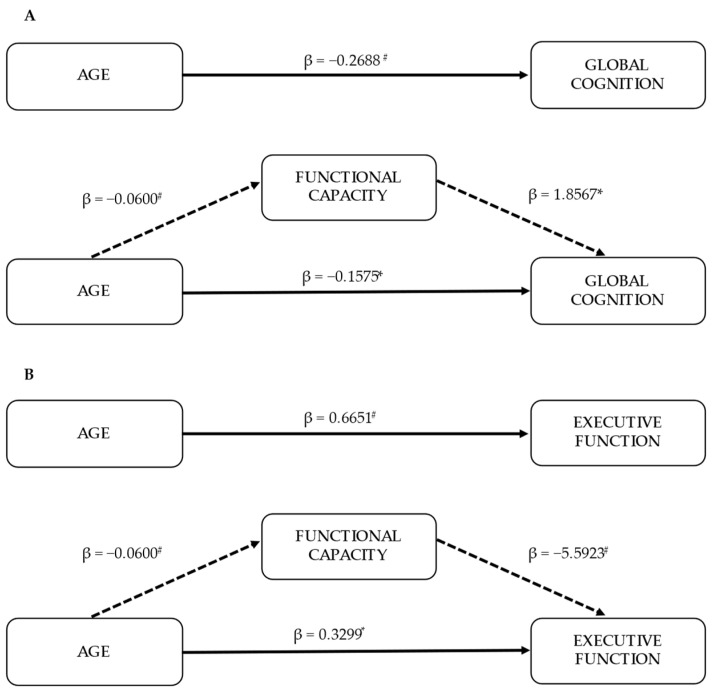
Mediation models (*n* = 89). Indirect effects of age on global cognition (as measured by MMSE score) (**A**) and executive function (as measured by errors in symbol cancellation test) (**B**) through functional capacity. Each model is controlled for sex and education. Standardized effects are presented. * *p* < 0.05; # *p* < 0.001.

Since functional capacity variables were significantly correlated with global cognition and executive function, it was examined whether functional capacity mediated the age-related effects of cognition. Therefore, a composite functional capacity score was derived by combining the six raw scores of functional capacity variables (6MWT, STS-5, STS-30, STS-60, TUG and HGS) and mediation analyses controlled for sex and education were performed. The mediation analysis revealed that functional capacity significantly partially mediated the effect of age on global cognition (indirect effect: β = −0.11, 95% CI = −0.20 to −0.03) (Figure 1A). A significant indirect effect of functional capacity was also found on the relationship between age and executive function (indirect effect: β = 0.34, 95% CI = 0.13 to 0.57) (Figure 1B).

## 4. Discussion

The results of the present study demonstrated that functional capacity could mediate the effect of aging on aspects related to global cognition and executive function. Greater levels of functional capacity were reflected in superior cognitive performance, higher quality of life, better sleep quality and lower fatigue levels in older adults, independent of confounding factors such as age, sex and education. These results highlight the importance of maintaining or even increasing the levels of functional capacity not only in the older population but throughout the human life span.

The positive association found in the present study between functional capacity and global cognitive function is consistent with previous studies [47,48,49,50]. Furthermore, even though all parameters of functional capacity correlated with global cognition, 6MWT presented the highest correlation, indicating the importance of preserving aerobic fitness in older adults. The results of the present study showed poor performance in 6MWT, which is probably due to the characteristics of the participants, such as advanced age, high obesity rates and that 75% of the sample were residents of long-term care facilities. Previous studies have shown that age and obesity are strong independent predictors of 6MWT performance [51]. Although the mechanisms underlying the aerobic fitness and cognitive function association have not been fully elucidated, previous observational studies have shown positive associations between levels of cardiorespiratory fitness and brain health factors related to cognition such as greater gray matter volume [52], hippocampal volume [53] and hippocampal effective connectivity [54]. Furthermore, longitudinal studies have also shown the influence of age on cognitive function and that results in functional capacity tests were significant predictors of changes in cognition [55,56]. Recent evidence suggests that poor performance in various functional capacity parameters can predict the incidence of dementia [57,58].

In addition, apart from global cognition, the present study aimed to investigate whether a specific cognitive domain such as the executive function could be related with various aspects of functional capacity. The symbol cancellation test, the measure of executive function examined in the current study, requires abilities such as neglect, visuospatial function, organizational process and selective attention [30,32,33], which are important for performing activities of daily living (ADL) [59] and instrumental activities of daily living (IADL) [22]. Even though only a few studies have examined the executive function–functional capacity relationship, the findings of the present study are consistent with previous results showing a positive association of aspects of executive function with various functional capacity parameters in older adults, such as cardiorespiratory fitness [49,53,60], handgrip and lower extremity strength [49,61,62]. Interestingly, in the present study, the observed association of functional capacity with executive function was higher than global cognition in almost all measured parameters, with the HGS presenting the strongest association. From prior literature, executive function appears to be more sensitive in functional decline than other cognitive domains or global measures of cognition [6,63], while executive dysfunction can be present without impairment on global cognitive status [63]. It has been previously suggested that exercise may have the greatest effect on executive function compared to other cognitive domains, as it may have a greater effect on the brain regions supporting executive processes such as the prefrontal cortex [64,65,66].

To further extend previous findings and to explore these aforementioned relationships, a mediation analysis was performed. In our model, functional capacity was evaluated as a potential mediator of the well-known age effect on cognitive functions. The results of the mediation analysis suggest that functional capacity can mediate the effects of age on both global cognition and executive function. Our results confirm the findings of one of the very few studies that performed a similar mediation analysis showing the mediator role of physical fitness on age-related global cognition decline [67]. However, that study examined physical fitness only by HGS and gait speed, while our study aimed to have a more holistic approach using a composite score of six functional capacity tests that are associated with different functional capacity parameters (e.g., 6MWT for endurance, STS-5 and HGS for lower and upper body strength, respectively, etc.). This ability of functional capacity provides an important pathway for delaying or even preventing cognitive impairment. Thus, increasing functional capacity through physical activity and exercise training can have a robust and beneficial influence on the brain health of older adults [13], with the largest fitness-induced benefits occurring for executive control processes [31,64]. This positive impact of increased functional capacity through physical activity and training on cognitive function during aging may work in multiple ways. Maintaining a higher level of functional capacity may reduce cerebrovascular, cardiovascular and metabolic risk factors that may largely impact cognitive function [13,26,68]. For example, chronic hypertension is a prominent risk factor for neurodegenerative diseases as it may induce structural and functional changes in cerebral blood vessels strongly affecting the cerebral circulation [26,69]. Research evidence suggests that age-related effects on cerebrovascular health and perfusion in older adults are largely influenced by their cardiorespiratory fitness levels [70].

As a cure for dementia has yet to be developed, early identification and intervention can be the most cost-effective ways to prevent or slow the onset of cognitive impairment and dementia. Therefore, this study provides beneficial information into the potential utility of functional capacity as a modifiable risk factor and a potential target for preventing cognitive decline in older adults, especially in those where polypharmacy seems to be an important barrier to effective multimorbidity management, thus reducing the rate of institutionalization and adverse outcomes in this age group. Therefore, future exercise interventions for cognitive aging should aim to improve functional capacity in order to minimize the deterioration of cognitive and executive functions associated with aging. Various training modalities such as aerobic, resistance and balance training have been shown to increase functional capacity [71,72,73], which may ultimately lead to improved cognitive function in older adults [74,75]. In this regard, a recent review argued that both physical and motor training modes may induce cognitive benefits, though via different pathways [76].

The positive association between functional capacity and quality of life that was found in the present study is also important to take into consideration. Low scores on both physical and mental health components were observed, with females presenting lower scores than male older adults, confirming previous research [77]. Interestingly, a recent review suggested that physical capability and quality of life are interlinked as exercise is effective at improving quality of life only when physical outcomes are improved [78]. This indicates the importance of various interventions in targeting functional capacity augmentation as an effective way to improve quality of life, especially in individuals with cognitive impairment [79,80]. Furthermore, all functional capacity parameters were associated with lower levels of fatigue. These results are of great importance as it has been suggested that fatigue is an early indicator of the aging process and a strong predictor of frailty, disability and mortality [16]. Moreover, around two-thirds of the participants presented low sleep quality. Poor sleep quality and duration are potential risk factors for dementia [26,81] and have been associated with low levels of quality of life and functional status [82], confirming our results. Notably, sleep quality was associated only with the aerobic and muscle endurance functional tests, indicating the beneficial impact of aerobic fitness on sleep quality [83].

The present study has some limitations. Firstly, only one executive function test was utilized; including tests of different executive subdomains may also be useful to parse aspects of executive function that are affected by the functional capacity at an individual level. In addition, while exploring a broader range of executive abilities, larger studies are needed to confirm our results. Furthermore, we cannot exclude reverse causation, i.e., that older participants with better cognitive function are more prone to engage in physical activity, thus having greater levels of functional capacity. These issues may be addressed further by longitudinal studies of this population. Finally, the results of the current study should be interpreted with caution as the majority of the participants were overweight/obese women, living in long-term care facilities. On the other hand, this study included a wide range of functional capacity parameters and explored these relationships across the continuum from normal aging to mild and moderate cognitive impairment.

## 5. Conclusions

This study showed that functional capacity mediates the deterioration of the cognitive and executive functions associated with aging. Furthermore, our results showed the positive associations of various functional capacity parameters with greater cognitive and executive functions and well-being in older adults. These results highlight the importance of maintaining or even increasing the levels of functional capacity in old age.

## Figures and Tables

**Table 1:** Sociodemographic characteristics, body composition, cognitive function and functional capacity results.

Variable	*n*	Overall	Male (*n* = 33)	Female (*n* = 64)
**Sociodemographic** **characteristics**				
Age (years)	97	80.6 ± 8.2	79.7 ± 7.2	81.0 ± 8.8
Charlson comorbidity index	97	2.3 ± 1.3	2.0 ± 1.4	2.4 ± 1.3
Education (years)	97	8.3 ± 4.4	10.4 ± 4.1	7.3 ± 4.2 ^#^
**Body composition**				
Body mass (kg)	89	69.6 ± 14.3	77.5 ± 14.4	64.9 ± 12.1 ^#^
BMI (kg/m^2^)	89	33.5 ± 5.8	33.6 ± 5.5	33.5 ± 6.0
Total body fat (%)	74	39.9 ± 8.6	32.5 ± 4.9	44.9 ± 6.8 ^#^
Trunk fat (%)	71	35.9 ± 10.1	31.2 ± 8.5	39.2 ± 9.8 ^#^
**Cognitive Function**				
MMSE score	97	21.5 ± 5.4	22.6 ± 4.7	20.9 ± 5.7
Symbol cancellation (errors)	97	46.1 ± 13.7	44.1 ± 13.6	47.1 ± 13.7
**Functional capacity**				
6MWT (m)	89	180.1 ± 164.8	211 ± 149.5	161.9 ± 171.8
STS-5 (s)	92	26.1 ± 14.5	19.6 ± 9.8	29.7 ± 15.5 ^#^
STS-30 (rep)	92	7.4 ± 4.0	8.8 ± 4.1	6.6 ± 3.7 *
STS-60 (rep)	92	12.6 ± 7.8	15.1 ± 7.6	11.3 ± 7.7 *
TUG (s)	89	27.2 ± 21.6	19.2 ± 16.0	32.0 ± 23.2 *
HGS (kgf)	91	17.8 ± 8.4	24.5 ± 8.5	14.0 ± 5.5 ^#^

* *p* < 0.05; # *p* < 0.001 between sexes; BMI: body mass index; MMSE: Mini-Mental State Examination; 6MWT: 6 min walk test; STS: sit-to stand test, TUG: timed up-and-go test; HGS: handgrip strength test.

**Table 2:** Scores of well-being questionnaires (quality of life, sleep quality, daytime sleepiness and fatigue) (*n* = 97).

Variable	Overall	Male (*n* = 33)	Female (*n* = 64)
**SF-36 Quality of life scales**			
Physical Function	36.5 ± 28.4	46.2 ± 27.3	31.6 ± 27.9 *
Role Physical	27.6 ± 38.3	31.8 ± 43.0	25.4 ± 35.8
Body Pain	60.8 ± 31.0	67.8 ± 30.5	57.1 ± 30.9
General Health	56.0 ± 21.7	62.2 ± 21.1	52.9 ± 21.5 *
Vitality	58.9 ± 24.7	63.8 ± 23.2	56.4 ± 25.3
Social Functioning	69.0 ± 26.1	69.9 ± 26.6	68.5 ± 26.0
Role Emotional	37.8 ± 45.6	56.5 ± 46.8	28.2 ± 42.1 *
Mental Health	62.4 ± 19.7	65.7 ± 20.1	60.7 ± 19.4
Physical Health Component	47.9 ± 21.3	54.2 ± 21.1	44.6 ± 20.8 *
Mental Health Component	56.8 ± 19.7	63.6 ± 19.4	53.3 ± 19.1 *
SF-36 total score	51.2 ± 19.6	58.0 ± 19.6	47.6 ± 18.9 *
**Sleep quality**	8.7 ± 5.0	8.0 ± 4.5	9.0 ± 5.3
**Daytime Sleepiness**	7.2 ± 4.9	7.3 ± 4.2	7.2 ± 5.2
**Fatigue**	4.4 ± 1.7	4.1 ± 1.8	4.5 ± 1.7

* p < 0.05 between sexes.

**Table 3:** Partial correlations between the measures of functional capacity, cognitive function and well-being, controlling for age, sex and education.

**Variables**		**1**	**2**	**3**	**4**	**5**	**6**	**7**	**8**	**9**	**10**	**11**	**12**
**1**	6MWT												
**2**	STS-5	−0.48 ^#^											
**3**	STS-30	0.66 ^#^	−0.73 ^#^										
**4**	STS-60	0.71 ^#^	−0.68 ^#^	0.93 ^#^									
**5**	TUG	−0.48 ^#^	0.59 ^#^	−0.52 ^#^	−0.53 ^#^								
**6**	HGS	0.53 ^#^	−0.30 *	0.47 ^#^	0.50 ^#^	−0.33 *							
**7**	MMSE	0.35 ^#^	−0.28 *	0.32 *	0.32 *	−0.27 *	0.23 *						
**8**	Symbol cancellation	−0.40 ^#^	0.35 ^#^	−0.43 ^#^	−0.40 ^#^	0.19	−0.49 ^#^	−0.48 ^#^					
**9**	Quality of life	0.38 ^#^	−0.34 *	0.40 ^#^	0.41 ^#^	−0.32 *	0.18	0.04	−0.10				
**10**	Sleep quality	−0.24 *	0.20	−0.23 *	−0.23 *	0.15	−0.08	−0.10	0.08	−0.54 ^#^			
**11**	Sleepiness	0.04	0.03	−0.07	−0.06	0.12	0.12	−0.13	−0.03	−0.31 *	0.15		
**12**	Fatigue	−0.37 ^#^	0.30 *	−0.33 *	−0.36 ^#^	0.35 *	−0.23 *	−0.06	0.22 *	−0.78 ^#^	0.43 ^#^	0.26 *	

* *p* < 0.05; # *p* < 0.001; 6MWT: 6 min walk test; STS: sit-to-stand test, TUG: timed up-and-go test; HGS: handgrip strength test; MMSE: Mini-Mental State Examination.

## Data Availability

The datasets used and/or analyzed during the current study are available from the corresponding author upon reasonable request.
